# Enhancing Early Childhood Mental Health Primary Care Services: Evaluation of MA Project LAUNCH

**DOI:** 10.1007/s10995-018-2548-4

**Published:** 2018-06-16

**Authors:** Beth E. Molnar, Kristin E. Lees, Kate Roper, Natasha Byars, Larisa Méndez-Peñate, Christy Moulin, William McMullen, Jessica Wolfe, Deborah Allen

**Affiliations:** 1 Institute on Urban Health Research, Northeastern University, 360 Huntington Ave., Boston, MA 02115 USA; 2 Boston Public Health Commission, 1010 Massachusetts Ave, Boston, MA 02118 USA; 3 Massachusetts Department of Public Health, 250 Washington St., Boston, MA 02118 USA; 4 Los Angeles County Department of Public Health, 313 N. Figueroa St., Los Angeles, CA 90012 USA; 5 Bouvé College of Health Sciences, Institute on Urban Health Research, Northeastern University, 360 Huntington Ave, M/S 314 INV, Boston, MA 02115 USA

**Keywords:** Early childhood, Mental health, LAUNCH, Primary care integration

## Abstract

*Objectives* The purpose of this study was to evaluate the efficacy of an innovative early childhood mental health intervention, Massachusetts Project LAUNCH. Early childhood mental health clinicians and family partners (paraprofessionals with lived experience) were embedded within community pediatric medical homes. *Methods* A longitudinal study design was used to test the hypotheses that (1) children who received services would experience decreased social, emotional and behavioral problems over time and (2) caregivers’ stress and depressive symptoms would decrease over time. Families who were enrolled in services and who consented to participate in the evaluation study were included in analyses (N = 225). Individual growth models were used to test longitudinal effects among MA LAUNCH participants (children and caregivers) over three time points using screening tools. *Results* Analyses showed that LAUNCH children who scored in age-specific clinically significant ranges of social, emotional and behavioral problems at Time 1 scored in the normal range on average by Time 3. Caregivers’ stress and depressive symptoms also declined across the three time points. Results support hypotheses that the LAUNCH intervention improved social and emotional health for children and caregivers. *Conclusions for Practice* This study led to sustainability efforts, an expansion of the model to three additional communities across the state and development of an online toolkit for other communities interested in implementation.

## Significance

Early childhood social-emotional difficulties are prevalent and can have negative impacts into adulthood. Integrating mental health services within primary care is an evidence-informed approach to addressing disparities in mental health care but efficacious models of reaching young children are needed. This study provides evidence that enhancing mental health services in primary care through the integration of trained early childhood mental health clinicians and family partners with lived experience can improve social-emotional health among young children and their caregivers.

## Introduction

An estimated 9–14% of children aged 0–5 are affected by social and emotional difficulties (Brauner and Bowers 2006) that limit ability to effectively engage in activities, fully benefit from educational opportunities (Zbar et al. 2016), avoid risky behaviors in adolescence (Thompson et al. 2011), and prevent emotional instability in adulthood (Goodman et al. 2011). Studies have identified multiple risk factors that affect early childhood mental health (ECMH) including neighborhood disadvantage, witnessing violence, parental emotional distress, mental illness or substance problems, incarcerated relatives or other caregiver separation, harsh discipline, and homelessness (Bayer et al. 2011; Garner et al. 2012). Timely interventions addressing barriers to social and emotional well-being are key to healthy development of children.

Literature on ECMH reflected in policy and practice recommendations (Foy 2010; American Academy of Child and Adolescent Psychiatry 2010), has identified burdens that insufficient mental health services place on medical providers, especially in practices serving low income children (Foy 2010). Primary care providers report low confidence in effectively screening and managing the mental health of young children, short visit time concerns, and hesitancy to refer to services with long wait times (Horwitz et al. 2007).

One suggested solution is improving the integration of mental health services into primary care (Ader et al. 2015; Foy 2010; AACAP 2010). Integration can take a variety of forms, including care coordination linked to mental health care, co-location of mental health and pediatric providers in same or nearby locations, or full integration of behavioral health specialists hired by primary care sites working iteratively with pediatric care providers about cases (Tyler et al. 2017). Integration has been more thoroughly explored in adult mental health care but is gaining traction in pediatrics (Godoy et al. 2014; Spijkers et al. 2013; Briggs et al. 2012). Early results are promising, suggesting improved identification of problems and improved screening results (Godoy et al. 2014; Spijkers et al. 2013; Briggs et al. 2012).

Needs of low income families often extend beyond children’s mental health services. A new approach is to provide more family-centered case coordination with help from “experience-based experts” (Gilkey et al. 2011). Family partners, one type of experience-based experts, have gained popularity in the movement toward patient and family-centered care for underserved, culturally, and linguistically diverse populations by community health care workers (Volkmann and Castanares 2011; American Academy of Pediatrics n.d.). Inclusion of experience-based experts has been associated with improved vaccination rates (Justvig et al. 2017), asthma control (Breysse et al. 2014), increased referrals to care coordination and improvements in patient support and education access (Matiz et al. 2014) among others. Family partners notably have a prominent role in evidence-based practices such as wrap-around care for children with serious emotional disorders (Bruns and Walker 2008). They typically have lived experiences with relevant systems of care; they can draw on shared experiences plus shared cultural and linguistic backgrounds to engage and educate families, and have been shown to improve family engagement, empowerment and trust in clinicians (Cournos and Goldfinger 2014).

This study presents results from the evaluation of Massachusetts’ Project Linking Actions with Unmet Needs in Children’s Health (MA_LAUNCH), a preventive intervention housed in community pediatric medical home settings. MA_LAUNCH addresses barriers to reducing ECMH risk through integration of an ECMH clinician and family partner team within pediatric primary care settings. This study presents evidence suggesting the efficacy of MA_LAUNCH in improving social, emotional and behavioral developmental progress of children and caregivers.

## Methods

### Study Design

A longitudinal, repeated measures study design was used to evaluate improvements in social, emotional, and behavioral development of children ages 0–8 years, and parenting-related stress and depressive symptoms of their caregivers, for participants in MA_LAUNCH. The evaluation ran from 2011 to 2015 at three treatment sites (N = 225 children). Children and caregivers were assessed at three time points: baseline (Time 1), 6 months (Time 2), and 12 months (Time 3) after enrollment. All families who received MA_LAUNCH services were eligible to participate in the study; records for those families who consented were transferred to the evaluation team for analyses. Two hypotheses were tested: (1) children would demonstrate improvements in social, emotional and behavioral development as measured by screening tools administered three times; and (2) parenting-related stress and depressive symptoms among caregivers would decrease over time.

### Intervention Design

MA_LAUNCH was conducted in Boston, Massachusetts and was designed to address needs of children at risk for social, emotional and behavioral problems by providing behavioral health services and case coordination within pediatric medical homes. The model (Boston Public Health Commission 2014) included the role of the “family partner,” as described above and below, to work collaboratively with a clinician who had masters-level training in mental health care for very young children. While the family partner role resembles that of other community health workers, it is distinguished in this model by the requirement that family partners have lived experience raising a child with a history of social, emotional or behavioral difficulties. The family partners were able to engage with families differently than the clinicians by drawing on shared experiences, modeling effective strategies for parenting, and advocating on behalf of their children. Both team members were employed by the health care site using funds from the grant and participated in ongoing trainings run jointly by the local and State public health departments on evidence-based early childhood development, mental health, and parenting interventions. At least one MA_LAUNCH team member at each site was multi-lingual in languages relevant to populations served. In addition to trainings, MA_LAUNCH teams benefitted from biannual cross-site/cross-project learning collaboratives and monthly meetings with medical and behavioral health staff from each site and the MA_LAUNCH team (Oppenheim et al. 2016). Clinical consultation, technical assistance and administrative supervision was provided by the local public health team throughout to assist in integration of MA_LAUNCH services into each center and in keeping fidelity to the model.

Massachusetts pediatric practices receiving MassHealth (Medicaid) reimbursement, including the three MA_LAUNCH sites, are required to implement behavioral screenings at each well-child visit. Based on screening results, clinician judgement or family concerns, warm handoffs were made to the MA_LAUNCH teams during the intervention period, who introduced families to the program and enrolled them if appropriate. Subsequent steps in the service delivery process were (1) completion of intake and informed consent processes, (2) administration of social and mental health needs assessments; (3) collaborative development of a care plan based on child needs and family priorities; (4) initiation of case management and related referrals; and, as needed, (5) child mental health and/or parenting interventions.

Multiple child and family factors were explored in clinical assessments—socioeconomic, relational, immigration, perinatal, traumatic, developmental, cultural, and more—and became important aspects of the team’s formulation and guiding framework for care plans. The in-depth clinical assessment included consultation with the primary care provider, objective screening tools, caregiver interviews gathering family history, observations of the child and family when possible, and play-based interaction when indicated; interventions were then tailored to the family’s unique needs. For example, a concern regarding a child’s behavior at childcare or preschool could stem from a variety of factors; after careful clinical assessment and formulation, such a concern might be addressed through a combination of developmental psychoeducation and guidance for the caregiver, observations at the school and/or consultation with the teacher, and follow-up as needed to provide the caregiver and/or teacher with recommendations. In multiple cases, support included helping the caregiver to better define and voice concerns with the school or daycare while simultaneously supporting the relationship between caregiver and school/teacher. This support often, in turn, strengthened the relationship with and availability of the school/teacher toward the child and became important to ameliorating the issue.

When adjustment was a concern, whether due to a new sibling, new schools, immigration, a separation or loss, or other cause, the team worked with the family, pairing play-based, dyadic intervention with caregiver guidance. Additionally, interventions often incorporated supporting the caregiver’s reflective functioning related to the child’s subjective experience, emotions, and responses, also known as parental mentalization. Parental mentalization is thought to not only support the child’s experience, regulation, and social emotional development, but to also support the caregiver’s own regulation and subjective experience as a parent in the face of challenges (Sharp and Fonagy 2008). Another significant opportunity presenting in primary care is seeing caregiver(s) during the perinatal period. Perinatal depression and emotional dysregulation represent the most common complications in the perinatal period and have impacts on the caregiver, baby, and family system (Meltzer-Brody 2011). Often complicated by additional stressors such as income loss and housing, perinatal mental health concerns were commonly referred to MA_LAUNCH teams by primary care providers and supporting families during this critical time became an area of focus. When child or family challenges necessitated interventions beyond the scope of MA_LAUNCH, the teams made referrals to in-house or external referral sources, but typically continued to be part of the family’s longer-term supportive pediatric care.

Trainings built into MA_LAUNCH benefited whole centers. For example, in one health center, 25 health center staff from diverse disciplines and roles—including primary care physicians, nurses, medical assistants, social workers, and interpreters—engaged in a two-day MA_LAUNCH-funded training on supporting budding relationships between caregivers and their newborns, and the caregiver’s own subjective sense of competency in their new role. In addition to these patient- and provider-specific activities, MA_LAUNCH teams developed activities within the medical home setting to engage and educate MA_LAUNCH families and overall pediatric clientele. Activities led by the family partners and clinicians from MA_LAUNCH were family-centered and encompassed both health promotion and prevention activities to engage whole families, including health center-wide events such as family game nights, kindergarten registration workshops, caregiver support groups, playgroups, and field trips.

For the evaluation study, assessment, screening and service data were entered by site teams into databases created by the evaluators. After families gave consent, these project records were transferred to the evaluators for analyses. Four Institutional Review Boards (IRB) reviewed and approved all procedures and protocols of the evaluation study: Northeastern University, MA Department of Public Health, Boston University and one participating site’s IRB.

### Participants

Three pediatric practices implemented MA_LAUNCH from 2010 to 2015; two were community health centers and one was a hospital-based pediatric clinic. All three served primarily low-income residents of surrounding Boston neighborhoods, where the majority of patients are families of color. All sites offered both pediatric and behavioral health care. Demographics of families are displayed in Table 1.

**Table 1:** Demographic characteristics of MA_LAUNCH evaluation study participants

Children N = 225	
Age	Mean (SD)
Age at intake—months	39.09 (24.07)
Age intake—years	3.26 (2.01)

	N (%)
Gender	
Male	140 (62)
Female	85 (38)
Race	
Biracial	10 (4)
Black	77 (34)
Hispanic	119 (53)
Multiracial	4 (2)
Other/unknown	5 (2)
White	9 (4)
Asian	1 (0.4)

Primary caregiver N = 186	
Age	Mean (SD)
Age at intake- years	30.19 (7.84)

	N (%)
Gender (2 missing)	
Male	4 (2)
Female	180 (97)
Race (6 missing)	
Biracial	1 (0.55)
Black/African American	70 (38)
Multiracial	3 (2)
White	11 (6)
Asian	2 (1)
Hispanic	84 (46)
Unknown	9 (5)

### Measures

Two parent-report screening tools were used to assess social emotional and behavioral concerns of children, with improvement defined as participant movement from clinically concerning ranges into healthy ranges. The Ages & Stages Social and Emotional questionnaire (ASQ-SE) was used with children age 5 and younger and the Child Behavior Checklist (CBCL) with children 6–8 years. For children who began the study at age 5 and ended at age 6, they were screened with the ASQ-SE first and the CBCL later. The ASQ-SE is a screening and monitoring tool to identify social emotional problems in children 0–6-years-old, with 19–30 items and good internal consistency (α = 0.82) and test–retest reliability (0.94) (Squires et al. 2001). Clinical cutoff scores for each age group indicate follow-up and monitoring needs (Squires et al. 2002). The CBCL contains 120 items assessing emotional or behavioral problems in the past 6 months, with scores above 63 considered clinically concerning; strong reliability and validity has been demonstrated in many populations (Achenbach and Rescorla 2008; Nakamura et al. 2008).

Two tools were used to measure caregiver functioning: the Patient Health Questionnaire-9 (PHQ-9) and the Parenting Stress Index-Short Form (3rd Edition) (PSI-SF). The PHQ-9 is a self-administered, nine-item depressive symptom subscale of the full PHQ based on DSM-IV diagnostic criteria, with demonstrated validity as a screening tool with diverse primary care patients, strong internal (α = 0.86–0.92) and test–retest (0.83–0.84) reliability (Huang et al. 2006), and 88% sensitivity/specificity for depression diagnoses (Kroenke et al. 2010). Each item is scored from 0 (not at all) to 3 (nearly every day) and summed to obtain scores from 0 to 27, with ≥10 representing clinically significant depressive symptoms. The PSI-SF is a 36-item abbreviated version of a 120-item scale used widely to measure overall parental stress (Abidin 1995). Scores from 16 to 84th percentiles are considered within the normal range while 85th percentile indicates clinically significant stress (Reitman et al. 2002).

### Statistical Analyses

Screening was done at the MA_LAUNCH sites across three time points. Individual growth modeling was used to analyze ASQ-SE and CBCL scores for MA_LAUNCH children, and the PSI and PHQ-9 scores for their caregivers. Multilevel models with restricted maximum likelihood estimations for mixed models were performed in SAS version 9.3. This procedure allows for the inclusion of all available data even for those growth records that were incomplete (Wolfinger and Chang 1995). Not all children and caregivers completed the screening tools at all time points (see figure captions for sample sizes); sensitivity analyses were conducted with subjects who completed two and three time points for each measure to confirm that growth model results were not biased by missing data.

## Results

### Social, Emotional and Behavioral Improvements for Children

**Fig. 1:**
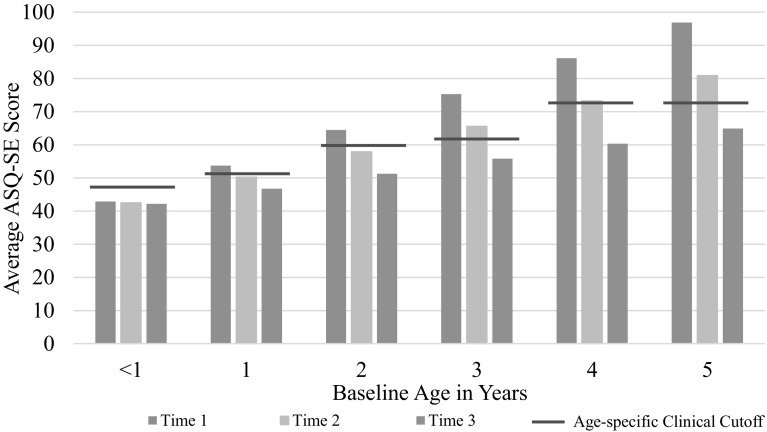
Changes in ASQ-SE scores over time (Time 1 N = 188; Time 2 N = 127; Time 3 N = 73) by age. ASQ-SE clinical cutoff scores vary by age and are indicated by the red line. Children aged 1–5 scored on average, above the cutoff scores at Time 1 and below the cutoff score at Time 3

Results from individual growth models (Table 2) assessing change in MA_LAUNCH children’s ASQ-SE scores over time (Fig. 1) show significant declines in social, emotional and behavioral problems for children under age 5 (N = 188 at baseline); children who started above the clinical cutoff score on average scored below (in the healthy range) by Time 3 (Χ^2^ = 74.73, p < 0.0001). Children ages 1–5 experienced a reduction in ASQ-SE scores putting them on average below the cutoff score by Time 3 (12-months post-enrollment), with the greatest change in children ages 4–5 years at baseline as indicated by a statistically significant (p = 0.03) age-time interaction estimate (− 3.13).

**Table 2:** Individual growth model results

Estimate (SE)				
Covariance Parameter	ASQ-SE	CBCL	PSI	PHQ-9
Individual	1032.62 (154.28)**	414.29(85.53)**	291.57 (44.76)**	12.97 (2.18)**
Residual	798.31(78.33)**	200.66(32.23)**	225.98 (21.11)**	14.98(1.37)**

Final models				
Parameter	ASQ-SE (Model X^2^ = 74.73 p < 0.0001)	CBCL (Model X^2^ = 46.22 p < 0.0001)	PSI (Model X^2^ = 46.92 p < 0.0001)	PHQ-9 (Model X^2^ = 15.41 p < 0.0001)
Intercept	42.90 (5.13)**	32.70 (2.57)**	65.71 (1.76)**	4.04 (0.33)**
Time	− 0.37 (4.51)	− 2.40 (1.72)	0.17 (1.19)	0.022 (0.27)
Age	10.80 (1.76)**			
Baseline risk		39.98 (4.86)**	34.64 (2.61)**	9.58 (0.61)**
Time*age	− 3.13 (1.47)*			
Time*baseline risk		− 10.86 (3.12)**	− 8.87 (1.84)**	− 2.94 (0.50)**

**p < 0.001; *p < 0.05

**Fig. 2:**
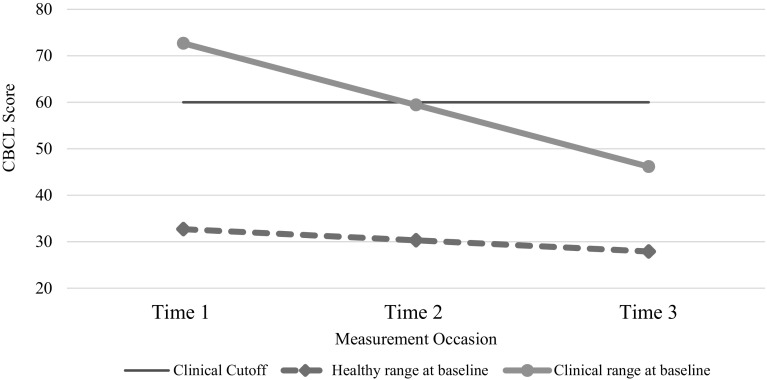
Comparison of children who scored above versus below the clinical cutoff score at Time 1 (Time 1 N = 75; Time 2 N = 45; Time 3 N = 28). On average, children who scored below the cutoff at baseline retained their healthy range status whereas children who scored above the cutoff at baseline, dropped below the cutoff by Time 3

For children ages 6–8 years (N = 75 at baseline), individual growth models were used to compare changes in CBCL total problems scores over the 1 year study period for children who scored above the clinical cutoff at Time 1 compared to children who scored in the healthy range at Time 1. While total problem scores of children who scored below the cutoff at Time 1 remained on average below the cutoff, the scores of children who scored above it at Time 1 dropped by over 25 points, a 37% decline (Fig. 2). The difference between the two groups was statistically significant as indicated by the time-baseline risk interaction estimate of − 10.86 (p < 0. 001).

### Stress and Depressive Symptoms Among Caregivers of MA_LAUNCH Children

**Fig. 3:**
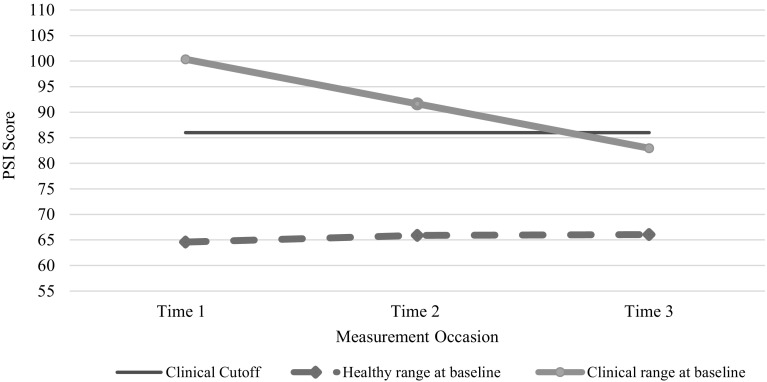
Comparison of caregivers who scored above versus below the clinical cutoff score at Time 1 (Time 1 N = 167; Time 2 N = 131; Time 3 N = 97). On average, caregivers who scored below the cutoff at baseline retained their healthy-range status whereas caregivers who scored above the cutoff at baseline, dropped below the cutoff by Time 3

Results from individual growth models of PSI-SF scores of MA_LAUNCH caregivers (N = 167 at baseline) compared caregivers with high parental stress levels at Time 1 (scores > 85) with caregivers within the healthy range (scores = 16–85) (Table 2; Fig. 3). Caregivers with scores above the cutoff showed an average decline of 17.38 points Time 1 to Time 3, bringing average scores for this group within the healthy range. Caregivers whose scores were within the healthy range did not change significantly Time 1–3. The difference between the groups was statistically significant as indicated by the time-baseline risk interaction estimate of − 8.87 (p < 0.001).

**Fig. 4:**
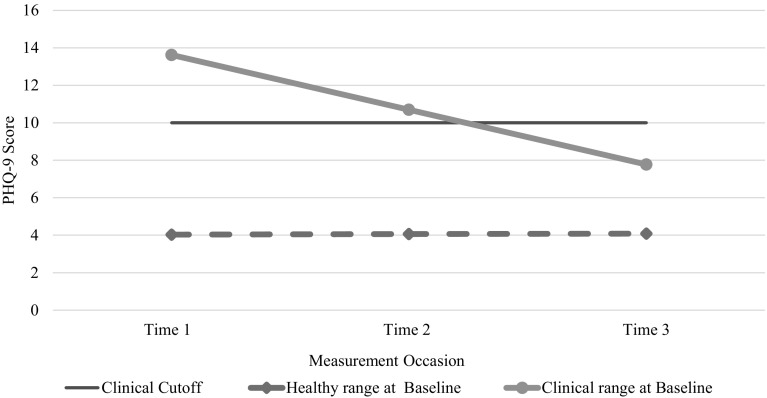
Comparison of caregivers who scored above versus below the clinical cutoff score at Time 1 (Time 1 N = 181; Time 2 N = 142; Time 3 N = 90). On average, caregivers who scored below the cutoff at baseline retained their healthy-range status whereas caregivers who scored above the cutoff at baseline, dropped below the cutoff by Time 3

For depressive symptoms as measured by the PHQ-9 (N = 181 caregivers at baseline), individual growth models compared MA_LAUNCH caregivers with clinical-level depressive symptoms at Time 1 (scores > 10) with those caregivers with scores in the non-clinical range. The fitted growth model (Table 2; Fig. 4) estimating change in depressive symptoms over time indicates that caregivers with high depressive symptoms at Time 1 had a decline of almost 6 points with scores on average falling within the non-clinical range by Time 3. Differences between the two groups were statistically significant as indicated by the time-baseline risk interaction estimate of − 2.94 (p < 0.001).

## Discussion

Children and caregivers served by MA_LAUNCH experienced significant improvements on common screening tools for social, emotional and behavioral health, especially for those participants with scores in clinically concerning ranges initially. Children had on average clinically significant screening results for all but the youngest age group upon study entry, and 1 year later they were (on average) in healthy ranges. Similarly, caregivers with clinically significant parenting-related stress and depressive symptom scores were on average below clinical cutoffs after 1 year. Children and caregivers whose screening results did not indicate clinical risk at the beginning of the study retained healthy status.

Evaluations of models that promote universal behavioral health screening and embed mental health clinicians within primary care sites reveal mixed results (Godoy et al. 2014; Spijkers et al. 2013; Briggs et al. 2012). For example, one model embedding an infant/toddler specialist in primary care settings reported improved ASQ-SE scores (Briggs et al. 2012) whereas one offering brief counseling found no improvements over usual care (Spijkers et al. 2013). Results from Project LAUNCH in Rhode Island, an initiative without the family partner role, resulted in improved identification of young children at risk but found that subsequently younger children were less likely than older children to utilize subsequent mental health consultation (Godoy et al. 2014). These results highlight needs for innovative family engagement approaches, particularly families with very young children in stressful circumstances.

The MA_LAUNCH intervention addresses gaps in ECMH service delivery for families by integrating trained family partners and mental health clinicians working as teams in primary care settings. MA_LAUNCH teams worked across sites in learning collaboratives, attended monthly meetings together, and received supervision from the state and local public health departments, facilitating sharing of resources and experiences. Situating the intervention within the medical home allowed families and health care professionals to maximize time together and make use of family partners’ shared experiences and cultural backgrounds. MA_LAUNCH teams promoted the medical home model’s ideal of family-centered approaches and confronted barriers to providers’ ability to address ECMH by providing immediate availability, support and center-wide activities for providers, families and to their clinics overall.

As with all studies, findings should be interpreted considering limitations. The first is that the ASQ-SE is designed to be used as a screening and monitoring tool rather than for diagnostic outcome measurement; this tool was used in this study to monitor children’s changes in problems over time. Results should not be interpreted as diagnostic. Secondly, for families in crisis, screenings were sometimes delayed until after the crisis subsided and thus after the family began receiving services. This delay may have resulted in underestimations of problems. Some subsequent assessments were also completed later than ideal due to scheduling difficulties. Some families were lost to follow up or did not complete assessments at all three time points. Reasons for loss to follow up included that the child aged out of MA_LAUNCH services, the family moved outside of the service area, or staff were unable to maintain contact with the family. However, sensitivity analyses showed the same results whether families completed two or three assessments. Sample size limited the researchers’ ability to include large numbers of covariates. This limitation was not expected to influence results due to the homogeneity of the sample; sensitivity analyses revealed no effect of excluded demographic variables. While a comparison site was recruited, budgetary and time constraints allowed for data collection only among children, and for only one follow-up screening assessment. Although the same criteria of exposure to a list of risk factors was utilized, very few children from the comparison group had ASQ-SE scores above the clinical cutoff at baseline. The lack of differences and limited data precluded meaningful comparisons of change between groups. Lack of meaningful comparison site data is a limitation of this study.

Given these evaluation results, future research examining the effectiveness of integrating behavioral health services into primary care is warranted, with attention on the impact of specific aspects of the MA_LAUNCH model. Although not included in the data presented here, process evaluation results showed the intervention to be widely accepted by caregivers who reported nearly unanimous satisfaction when interviewed by the evaluation team as to whether the MA_LAUNCH team helped them understand their children’s emotions and behaviors and whether they helped them help their children express feelings in more positive ways. Data collected from non-LAUNCH providers within the clinical sites and the MA_LAUNCH teams themselves also reported many benefits including care coordination successes, integration of services, and increased access to school registration and school and child care services. Components to explore further include whether dose–response effects exist for families who receive services more frequently or for longer periods of time; impacts of the family partner role versus the clinician role; the influence of full integration of the model into primary care on service acceptance and adherence; and physicians’ perceived abilities to incorporate behavioral health treatment into their practice.

Project LAUNCH in Massachusetts was delivered in community-based pediatric primary care practices in low income urban settings. Children served at these centers are primarily recipients of MassHealth, the state Medicaid program. Families in this study experienced community- and family-level risk factors, and health and/or education disparities. The results of the evaluation of the MA_LAUNCH project show that both children and caregivers experienced improvements in social, emotional and behavioral difficulties. Given these results, it is likely MA_LAUNCH helped to overcome barriers to service delivery and attainment of social and emotional well-being. Interventions are needed that put children on a healthy developmental pathway. The results of this study can be used to inform the development of similar programs and policies to address disparities in access to behavioral health care and well-being for very young children.

## References

[CR1] Abidin RR (1995). Parenting stress index, third edition: professional manual.

[CR2] Achenbach T, Rescorla L (2008). Manual for the ASEBA School-Age Forms & Profiles: an integrated system of multi-informant assessment.

[CR3] Ader J, Stille CJ, Keller D, Miller BF, Barr MS, Perrin JM (2015). The medical home and integrated behavioral health: Advancing the policy agenda. Pediatrics.

[CR4] American Academy of Child and Adolescent Psychiatry. (2010). A guide to building collaborative mental health care partnerships in pediatric primary care. Retrieved March 15, 2017, from https://www.aacap.org/App_Themes/AACAP/docs/clinical_practice_center/guide_to_building_collaborative_mental_health_care_partnerships.pdf.

[CR5] American Academy of Pediatrics. (n.d.). Salud para todos improving health through medical homes. Retrieved from https://medicalhomeinfo.aap.org/tools-resources/Documents/RI%20FactSheet.pdf.

[CR6] Bayer JK, Ukoumunne OC, Lucas N, Wake M, Scalzo K, Nicholson JM (2011). Risk factors for childhood mental health symptoms: National longitudinal study of Australian children. Pediatrics.

[CR7] Boston Public Health Commission. (2014). Early childhood mental health toolkit: Integrating mental health services into the pediatric medical home. Retrieved March 15, 2017, from http://www.ecmhmatters.org/ForProfessionals/Pages/MedicalHome.aspx.

[CR8] Brauner BC, Bowers SC (2006). Estimating the prevalence of early childhood serious emotional/behavioral disorders: Challenges and recommendations. Public Health Reports.

[CR9] Breysse J, Dixon S, Gregory J, Philby M, Jacobs DE, Krieger J (2014). Effect of weatherization combined with community health worker in-home education on asthma control. American Journal of Public Health.

[CR10] Briggs RD, Stettler E, Silver E, Schrag R, Nayak M, Chinitz S, Racine AD (2012). Social-emotional screening for infants and toddlers in primary care. Pediatrics.

[CR11] Bruns, E. J., & Walker, J. S. (Eds.) (2008). The resource guide to wraparound. Portland, OR: National Wraparound Initiative, Research and Training Center for Family Support and Children’s Mental Health. Retrieved March 15, 2017, from https://nwi.pdx.edu/NWI-book/Chapters/COMPLETE-RG-BOOK.pdf.

[CR12] Cournos F, Goldfinger SM (2014). Family partners improve early childhood mental health services. Psychiatric Services.

[CR13] Foy JM (2010). Enhancing pediatric mental health care: Report from the American Academy of Pediatrics Task Force on Mental Health. Introduction. Pediatrics.

[CR14] Garner AS, Shonkoff JP, Siegel BS, Dobbins MI, Earls MF, Garner AS, Wood DL (2012). Early childhood adversity, toxic stress, and the role of the pediatrician: Translating developmental science into lifelong health. Pediatrics.

[CR15] Gilkey M, Garcia CC, Rush C (2011). Professionalization and the experience-based expert: Strengthening partnerships between health educators and community health workers. Health Promotion Practice.

[CR16] Godoy L, Carter AS, Silver RB, Dickstein S, Seifer R (2014). Mental health screening and consultation in primary care: The role of child age and parental concerns. Journal of Devevelopmental and Behavior Pediatrics.

[CR17] Goodman A, Joyce R, Smith JP (2011). The long shadow cast by childhood physical and mental problems on adult life. Proceedings of the National Academy Sciences of the United States of America.

[CR18] Horwitz SM, Kelleher KJ, Stein RE, Storfer-Isser A, Youngstrom EA, Park ER, Hoagwood KE (2007). Barriers to the identification and management of psychosocial issues in children and maternal depression. Pediatrics.

[CR19] Huang F, Chung H, Kroenke K, Delucchi K, Spitzer R (2006). Using the Patient Health Questionnaire-9 to measure depression among racially and ethnically diverse primary care patients. Journal of General Internal Medicine.

[CR20] Justvig SP, Li J, Caravella G, Chen M, Wang H, Benz Scott LA, Pati S (2017). Improving adherence to care recommendations using a community health worker (CHW) intervention with the pediatric medical home. Journal of Community Health.

[CR21] Kroenke K, Spitzer RL, Williams JBW, Löwe B (2010). The patient health questionnaire somatic, anxiety, and depressive symptom scales: A systematic review. General Hospital Psychiatry.

[CR22] Matiz LA, Peretz PJ, Jacotin PG, Cruz C, Ramirez-Diaz E, Nieto A (2014). The impact of integrating community health workers into the patient-centered medical home. Journal of Primary Care and Community Health.

[CR23] Meltzer-Brody S (2011). New insights into perinatal depression: Pathogenesis and treatment during pregnancy and postpartum. Dialogues in Clinical Neuroscience.

[CR24] Nakamura BJ, Ebesutani C, Bernstein A, Chorpita BF (2008). A psychometric analysis of the child behavior checklist DSM-oriented scales. Journal of Psychopathology and Behavioral Assessment.

[CR25] Oppenheim J, Stewart W, Zoubak E, Donato I, Huang L, Hudock W (2016). Launching forward: The integration of behavioral health in primary care as a key strategy for promoting young child wellness. American Journal of Orthopsychiatry.

[CR26] Reitman D, Currier RO, Stickle T (2002). A critical evaluation of the parenting stress index-short form (PSI-SF) in a Head Start population. Journal of Clinical Child and Adolescent Psychology.

[CR27] Sharp C, Fonagy P (2008). The parent’s capacity to treat the child as a psychological agent: Constructs, measures and implications for developmental psychopathology. Social Development.

[CR28] Spijkers, W., Jansen, D. E., & Reijneveld, S. A. (2013). Effectiveness of primary care Triple P on child psychosocial problems in preventive child healthcare: A randomized controlled trial.* BMC Medicine*. 10.1186/1741-7015-11-240. 10.1186/1741-7015-11-240 PMC4226010 24207163

[CR29] Squires J, Bricker D, Heo K, Twombly E (2001). Identification of social-emotional problems in young children using a parent-completed screening measure. Early Childhood Research Quarterly.

[CR30] Squires J, Bricker D, Twombly E (2002). The ASQ:SE user’s guide: For the Ages & Stages Questionnaires: Social-emotional.

[CR31] Thompson R, Tabone JK, Litrownik AJ, Briggs EC, Hussey JM, English DJ, Howard D (2011). Early adolescent risk behavior outcomes of childhood externalizing behavioral trajectories. The Journal of Early Adolescence.

[CR32] Tyler, E. T., Hulkower, R. L., & Kaminski, J. W. (2017). Milbank memorial fund. Behavioral health integration in pediatric primary care: Considerations and opportunities for policymakers, planners, and providers. Retrieved March 15, 2017, from https://www.milbank.org/publications/behavioral-health-integration-in-pediatric-primary-care-considerations-and-opportunities-for-policymakers-planners-and-providers/.

[CR33] Volkmann K, Castanares T (2011). Clinical community health workers: linchpin of the medical home. The Journal of Ambulatory Care Management.

[CR34] Wolfinger, R., & Chang, M. (1995) Comparing the SAS® GLM and MIXED procedures for repeated measurements analysis. Retrieved March 15, 2017, from https://support.sas.com/rnd/app/stat/papers/abstracts/mixedglm.html.

[CR35] Zbar A, Surkan PJ, Fombonne E, Melchior ME (2016). Emotional and behavioral difficulties and adult educational attainment: An 18-year follow-up of the TEMPO study. European Child and Adolescent Psychiatry.

